# Supplementary material for: Genome-Wide Identification and Analysis of bHLH Transcription Factors Related to Anthocyanin Biosynthesis in Cymbidium ensifolium
Source: Int J Mol Sci. 2023 Feb 14;24(4):3825. doi: 10.3390/ijms24043825 (PMC9963586; doi:10.3390/ijms24043825)
Supplement: Supplementary file 1 [file ijms-24-03825-s001.zip › ijms-2196314-supplementary.pdf]

Supplementary materials

Table S1. The characteristics of 94 CebHLHs

Gene name	Gene ID	Chromosome	Number of amino acid (aa)	CDS length (bp)	Molecular weight (Da)	Isoelectric point (*pI*)	Aliphatic index	Grand average of hydropathicity (GRAVY)	Subcellular localization
CebHLH1	JL008540	Chr01	383	1152	41114.77	8.87	70.65	-0.433	Nuclear
CebHLH2	JL016410	Chr01	647	1944	71192.78	6.76	69.97	-0.554	Nuclear
CebHLH3	JL007713	Chr01	390	1173	42757.24	5.98	85.97	-0.280	Nuclear
CebHLH4	JL014883	Chr01	260	783	29687.83	9.77	81.42	-0.683	Nuclear
CebHLH05	JL024782	Chr01	252	759	28848.80	9.56	81.31	-0.617	Nuclear
CebHLH06	JL013196	Chr01	186	561	20844.52	5.21	80.11	-0.498	Cytoplasmic
CebHLH07	JL008801	Chr01	275	828	30015.81	5.92	88.44	-0.296	Nuclear
CebHLH08	JL001269	Chr01	251	756	28157.07	5.93	76.57	-0.490	Nuclear
CebHLH09	JL000319	Chr01	253	762	27830.68	10.13	81.50	-0.310	Cytoplasmic
CebHLH10	JL006798	Chr02	249	750	27017.66	7.69	85.82	-0.255	Nuclear
CebHLH11	JL009723	Chr02	363	1092	39664.88	6.03	79.92	-0.400	Nuclear
CebHLH12	JL011575	Chr03	764	2295	83816.05	6.22	68.30	-0.504	Nuclear
CebHLH13	JL010983	Chr03	687	2064	78308.27	5.73	84.56	-0.526	Nuclear
CebHLH14	JL009396	Chr03	575	1728	63845.53	5.08	74.85	-0.510	Chloroplast
CebHLH15	JL001320	Chr03	346	1041	38463.77	5.50	73.06	-0.573	Nuclear
CebHLH16	JL010236	Chr03	79	240	9101.48	9.76	93.80	-0.294	Chloroplast
CebHLH17	JL010652	Chr03	85	258	9535.81	6.58	94.24	-0.525	Nuclear
CebHLH18	JL001467	Chr03	305	918	34448.13	9.10	84.46	-0.445	Nuclear
CebHLH19	JL020648	Chr03	197	594	20885.74	9.06	88.17	-0.184	Nuclear

CebHLH20	JL024671	Chr03	174	525	19095.90	9.37	92.59	-0.233	Nuclear
CebHLH21	JL026773	Chr03	122	369	13395.62	10.72	99.92	-0.181	Cytoplasmic
CebHLH22	JL007671	Chr03	273	822	31034.53	5.79	86.45	-0.355	Cytoskeleton
CebHLH23	JL015016	Chr03	89	270	10054.29	6.58	88.88	-0.655	Nuclear
CebHLH24	JL008345	Chr04	312	939	34680.19	5.43	77.82	-0.464	Nuclear
CebHLH25	JL011143	Chr04	466	1401	50991.95	5.44	66.39	-0.591	Nuclear
CebHLH26	JL002683	Chr04	328	987	36406.06	9.16	74.05	-0.663	Nuclear
CebHLH27	JL007424	Chr04	306	921	34590.41	6.13	82.78	-0.412	Nuclear
CebHLH28	JL000425	Chr04	221	666	24682.83	9.34	69.37	-0.662	Nuclear
CebHLH29	JL019573	Chr04	144	435	16036.70	8.97	99.58	-0.200	Chloroplast
CebHLH30	JL022231	Chr04	267	804	29511.22	8.67	69.78	-0.591	Chloroplast
CebHLH31	JL012023	Chr04	500	1503	55218.66	5.23	89.52	-0.168	Nuclear
CebHLH32	JL010823	Chr04	213	642	24160.83	9.04	94.79	-0.410	Cytoplasmic
CebHLH33	JL017021	Chr05	497	1494	53492.63	5.73	75.19	-0.436	Nuclear
CebHLH34	JL010661	Chr05	205	618	22577.58	7.94	66.68	-0.409	Nuclear
CebHLH35	JL017952	Chr05	226	681	24761.71	7.65	78.23	-0.705	Chloroplast
CebHLH36	JL002881	Chr06	418	1257	45868.72	6.32	84.67	-0.363	Nuclear
CebHLH37	JL002882	Chr06	190	573	21259.48	9.67	95.89	-0.465	Nuclear
CebHLH38	JL013595	Chr06	530	1593	56711.89	5.06	78.08	-0.303	Nuclear
CebHLH39	JL016769	Chr06	425	1278	46848.45	6.81	69.76	-0.616	Nuclear
CebHLH40	JL004230	Chr06	294	885	33390.64	6.23	79.59	-0.615	Nuclear
CebHLH41	JL025976	Chr06	243	732	28019.26	9.58	73.79	-0.750	Chloroplast
CebHLH42	JL001783	Chr06	401	1206	44188.63	6.74	84.06	-0.346	Endoplasmic reticulum
CebHLH43	JL025962	Chr07	602	1809	65863.34	8.75	72.21	-0.496	Nuclear
CebHLH44	JL003257	Chr07	320	963	35395.15	6.13	83.56	-0.358	Chloroplast

CebHLH45	JL003229	Chr07	421	1266	46044.95	5.89	85.04	-0.312	Nuclear
CebHLH46	JL015540	Chr07	381	1146	41652.03	6.34	81.65	-0.377	Nuclear
CebHLH47	JL005271	Chr07	711	2136	77851.65	6.00	71.66	-0.455	Nuclear
CebHLH48	JL022460	Chr07	357	1074	38886.80	6.27	58.74	-0.830	Nuclear
CebHLH49	JL014332	Chr08	361	1086	40096.83	8.77	58.92	-0.724	Nuclear
CebHLH50	JL009633	Chr08	315	948	34579.33	6.54	64.48	-0.805	Nuclear
CebHLH51	JL009635	Chr08	365	1098	39737.09	6.15	66.11	-0.693	Nuclear
CebHLH52	JL009179	Chr08	225	678	25776.82	5.26	67.69	-0.704	Nuclear
CebHLH53	JL014528	Chr09	255	768	28714.62	7.66	83.80	-0.453	Nuclear
CebHLH54	JL005537	Chr09	432	1299	48551.78	9.34	93.45	-0.344	Nuclear
CebHLH55	JL006708	Chr09	229	690	25839.16	4.81	74.02	-0.472	Nuclear
CebHLH56	JL019946	Chr09	259	780	29251.35	4.83	69.03	-0.754	Nuclear
CebHLH57	JL014263	Chr09	314	945	34357.05	6.03	93.79	-0.249	Nuclear
CebHLH58	JL016976	Chr10	268	807	29118.45	5.93	69.59	-0.611	Nuclear
CebHLH59	JL010513	Chr10	444	1335	45714.06	5.71	72.91	-0.277	Nuclear
CebHLH60	JL004493	Chr10	210	633	23119.94	9.90	86.86	-0.253	Chloroplast
CebHLH61	JL000658	Chr10	392	1179	42218.12	7.63	66.53	-0.460	Nuclear
CebHLH62	JL025659	Chr10	253	762	27910.15	5.48	85.30	-0.101	Nuclear
CebHLH63	JL018932	Chr10	330	993	36311.62	5.15	62.39	-0.694	Cytoplasmic
CebHLH64	JL005824	Chr11	174	525	19759.61	8.88	93.05	-0.567	Cytoplasmic
CebHLH65	JL025300	Chr11	169	510	18902.99	6.16	99.17	-0.176	Nuclear
CebHLH66	JL004518	Chr11	298	897	34035.25	6.87	69.97	-0.726	Nuclear
CebHLH67	JL009855	Chr11	559	1680	61612.65	6.12	76.21	-0.424	Nuclear
CebHLH68	JL005362	Chr11	375	1128	41054.24	4.85	82.51	-0.358	Nuclear
CebHLH69	JL004531	Chr11	452	1359	48769.82	6.34	69.51	-0.492	Nuclear
CebHLH70	JL007774	Chr11	265	798	28501.37	9.88	77.02	-0.418	Mitochondrial

CebHLH71	JL006752	Chr11	315	948	35154.04	4.95	69.68	-0.673	Nuclear
CebHLH72	JL021099	Chr12	264	795	29134.49	7.94	67.58	-0.734	Nuclear
CebHLH73	JL012150	Chr12	309	930	34701.55	5.70	88.38	-0.361	Nuclear
CebHLH74	JL007736	Chr12	482	1449	51990.31	5.40	70.89	-0.481	Nuclear
CebHLH75	JL019154	Chr13	670	2013	76096.66	5.39	78.42	-0.507	Nuclear
CebHLH76	JL009274	Chr13	352	1059	37730.85	6.71	62.90	-0.575	Chloroplast
CebHLH77	JL024438	Chr14	437	1314	48689.66	5.69	68.31	-0.468	Nuclear
CebHLH78	JL006337	Chr14	292	879	32929.35	5.09	77.53	-0.508	Nuclear
CebHLH79	JL015771	Chr14	244	735	27400.49	7.66	70.00	-0.689	Nuclear
CebHLH80	JL006914	Chr14	279	840	31910.77	7.07	89.43	-0.384	Nuclear
CebHLH81	JL013113	Chr15	85	258	9724.01	6.58	90.71	-0.694	Chloroplast
CebHLH82	JL016513	Chr15	316	951	35167.00	6.03	83.39	-0.284	Nuclear
CebHLH83	JL009988	Chr15	332	999	35974.45	5.25	84.16	-0.389	Nuclear
CebHLH84	JL000223	Chr15	233	702	26192.80	6.06	70.39	-0.715	Nuclear
CebHLH85	JL021345	Chr16	326	981	35994.65	5.85	75.67	-0.478	Cytoskeleton
CebHLH86	JL022359	Chr16	416	1251	45592.88	5.76	76.23	-0.522	Nuclear
CebHLH87	JL003657	Chr16	527	1584	56829.94	5.17	78.88	-0.270	Nuclear
CebHLH88	JL000551	Chr17	291	876	32244.89	4.79	77.22	-0.335	Nuclear
CebHLH89	JL015227	Chr17	314	945	35051.91	8.81	82.61	-0.299	Nuclear
CebHLH90	JL022349	Chr17	143	432	16043.67	8.97	91.47	-0.325	Chloroplast
CebHLH91	JL025286	Chr17	144	435	16239.96	9.26	94.17	-0.336	Integral membrane protein
CebHLH92	JL003085	Chr18	388	1167	44542.75	9.70	85.88	-0.279	Mitochondrial
CebHLH93	JL003086	Chr18	334	1005	38421.51	9.45	84.04	-0.216	Nuclear
CebHLH94	JL003888	Chr19	477	1434	49851.35	8.60	79.04	-0.236	Chloroplast

Table S2. *Ka/Ks* analysis of *CebHLHs*

Seq_1	Seq_2	*Ka*	*Ks*	*Ka/Ks*	Duplication type	Purify selection
CebHLH11	CebHLH57	0.223524312	1.302055357	0.17167036	Segmental	Yes
CebHLH13	CebHLH75	0.227973347	0.781712024	0.291633415	Segmental	Yes
CebHLH17	CebHLH81	0.059437029	1.09576343	0.054242574	Segmental	Yes
CebHLH18	CebHLH80	0.364059191	1.890845935	0.192537734	Segmental	Yes
CebHLH18	CebHLH82	0.299750451	1.236719136	0.242375526	Segmental	Yes
CebHLH2	CebHLH47	0.181453151	0.991036454	0.183094325	Segmental	Yes
CebHLH25	CebHLH26	0.229468701	0.74493883	0.308036971	Segmental	Yes
CebHLH30	CebHLH69	0.474070668	2.041840307	0.232178132	Segmental	Yes
CebHLH36	CebHLH45	0.186989915	1.715684589	0.108988515	Segmental	Yes
CebHLH45	CebHLH46	0.250912182	1.863225855	0.134665468	Segmental	Yes
CebHLH56	CebHLH71	0.241994403	0.844209668	0.286652016	Segmental	Yes
CebHLH59	CebHLH94	0.190556796	0.992500292	0.191996715	Segmental	Yes
CebHLH80	CebHLH82	0.298098194	2.417854303	0.123290387	Segmental	Yes
CebHLH20	CebHLH21	0.030135881	0.021315822	1.413779904	Tandem	NO
CebHLH36	CebHLH37	0.002355715	NaN	NaN	Tandem	/
CebHLH4	CebHLH5	0.051343936	0.044741124	1.14757814	Tandem	NO
CebHLH41	CebHLH42	0.118644252	0.218042525	0.544133545	Tandem	Yes
CebHLH50	CebHLH51	0.009902768	0.025795127	0.383900727	Tandem	Yes
CebHLH92	CebHLH93	0.023796766	0.054342888	0.437900292	Tandem	Yes

Table S3. The FPKM values of *CebHLHs* of different color sepals of *C. ensifolium*

Gene name	Gene ID	Purple-red	Red	Yellow-green	White
CebHLH1	JL008540	0.985129405	1.504891525	9.610858258	1.872601368
CebHLH2	JL016410	20.50920223	6.242717396	6.91440287	7.350021815
CebHLH4	JL014883	14.61330139	26.95219472	5.386000471	43.99939438
CebHLH5	JL024782	20.91594336	10.17476971	5.970739059	17.17261533
CebHLH6	JL013196	0.034514284	0	0	0
CebHLH7	JL008801	3.071626443	10.57155369	6.249639627	5.922564841
CebHLH8	JL001269	2.919659594	5.089479472	2.471100741	6.058462725
CebHLH9	JL000319	5.887904786	3.185729263	2.296451975	2.672551885
CebHLH10	JL006798	24.62939242	16.36834815	22.27332069	17.88289627
CebHLH11	JL009723	3.594761359	2.470469837	1.738415613	5.214398542
CebHLH12	JL011575	83.59337844	35.05169088	93.42667369	62.96261948
CebHLH13	JL010983	50.68071839	17.68106156	39.64624922	28.90844981
CebHLH14	JL009396	0.13944284	0.043332681	0	0.015986198
CebHLH15	JL001320	0.249850475	0.554015045	0.07386416	0.295269221
CebHLH17	JL010652	6.187773035	6.430547752	0.202133137	20.28468527
CebHLH18	JL001467	0	0.027189133	0	0.030283032
CebHLH19	JL020648	0.419472066	0.411736207	0.041653604	0.0936021
CebHLH22	JL007671	0.681490294	0.179206975	0.061821673	0.286453795
CebHLH23	JL015016	237.8917561	553.765711	164.3378818	742.4865307
CebHLH24	JL008345	8.209123316	4.222228446	1.947276962	7.580347155
CebHLH25	JL011143	97.86315439	204.5501466	47.89674747	133.9858809
CebHLH26	JL002683	164.6637788	309.7562618	106.9305561	236.2534142
CebHLH27	JL007424	0.650339796	0.027100569	0.081648013	0.134924783
CebHLH28	JL000425	0.487379842	0.069590817	0.300849355	0.170389171

CebHLH30	JL022231	8.434826867	13.04838516	7.871792775	18.95992902
CebHLH31	JL012023	13.53463496	2.921141818	3.054374414	0.864184836
CebHLH33	JL017021	50.71098062	50.05944731	63.71386637	57.91100442
CebHLH34	JL010661	0.946663359	0.767094528	0.665872529	1.447129374
CebHLH35	JL017952	18.34388956	12.5972863	25.43571351	31.28411233
CebHLH36	JL002881	5.077663618	7.620129772	22.36818873	6.786385116
CebHLH37	JL002882	0.260749887	1.723248935	0.985704807	0.313901173
CebHLH38	JL013595	4.266622988	2.343125058	1.417423822	1.331570547
CebHLH39	JL016769	17.50721471	13.1235904	20.90380204	17.86031869
CebHLH43	JL025962	10.42271529	9.788716573	21.37864715	18.85395548
CebHLH44	JL003257	0.44042191	0.263455575	2.988380105	1.774958671
CebHLH45	JL003229	0.895102588	3.189978054	0.626721974	4.511499414
CebHLH46	JL015540	28.54268726	54.64726034	100.0350582	135.0521964
CebHLH47	JL005271	74.71822752	60.49751157	48.53281838	42.03452085
CebHLH48	JL022460	15.34488263	13.36085436	11.88609974	15.87643824
CebHLH49	JL014332	2.309695125	0.872631648	1.885270031	0.78781208
CebHLH50	JL009633	4.43066071	5.143506899	4.594098861	4.945951494
CebHLH51	JL009635	14.59262118	9.330921216	9.304078397	10.86162699
CebHLH52	JL009179	0.115593988	0.036813605	0	0.08200538
CebHLH54	JL005537	0	0.090573204	0	0.110514939
CebHLH55	JL006708	0.028061613	0.037617486	0	0
CebHLH56	JL019946	0.141903409	0.227990263	0.223913246	0.166847599
CebHLH57	JL014263	0	0.185324201	0.052364531	0
CebHLH58	JL016976	128.0823017	288.5363963	157.2991048	296.1211414
CebHLH59	JL010513	46.62750238	15.55777789	26.9056451	11.67332763
CebHLH60	JL004493	0	0.041004843	0	0.030478958

CebHLH61	JL000658	74.01341628	32.16559133	49.03650128	37.26400011
CebHLH62	JL025659	0	0.034063078	0	0
CebHLH63	JL018932	1.167319209	0.219026272	0.128734862	0.161527209
CebHLH64	JL005824	64.79763579	55.30840685	105.1412564	31.0284424
CebHLH65	JL025300	2.475926016	6.753782442	7.223867795	4.901365739
CebHLH66	JL004518	1.962490701	0.521325576	10.24155697	0.477880039
CebHLH67	JL009855	17.2407045	7.200106272	11.41148848	8.624508517
CebHLH68	JL005362	0.01926652	0.115053482	0.022365062	0.049134728
CebHLH69	JL004531	1.027694089	0.929672416	0.360721336	0.760612622
CebHLH70	JL007774	0.497809374	5.391999216	0.746075664	3.682802029
CebHLH72	JL021099	3.36852141	8.425013747	3.63669741	2.226492365
CebHLH73	JL012150	0.020819907	0.455760087	0.02660456	0.020745355
CebHLH74	JL007736	12.74864345	9.857597246	13.49787599	30.90247544
CebHLH75	JL019154	2.444778093	7.36556776	0.538280093	0.569394605
CebHLH76	JL009274	7.518681951	16.66844299	14.72075198	16.17601495
CebHLH77	JL024438	3.598319896	4.600355367	8.684345553	4.405631896
CebHLH78	JL006337	1.053187606	0.347690386	1.039676218	0.909085213
CebHLH79	JL015771	5.939076162	3.461516808	2.133616687	2.333434971
CebHLH80	JL006914	1.856781306	1.910873311	3.658589422	2.614911704
CebHLH81	JL013113	4.058869605	1.537901788	0.095900158	7.474572203
CebHLH82	JL016513	3.59267889	2.028292745	1.705108868	1.621227589
CebHLH83	JL009988	0.323245372	0.305925775	0.149574117	0.537588364
CebHLH84	JL000223	121.9049607	272.787499	260.2628966	226.4729546
CebHLH85	JL021345	0.161612166	0.294393675	0	0.276856079
CebHLH86	JL022359	21.29866653	147.8510665	53.66366486	136.1961303
CebHLH87	JL003657	0.766849273	3.559083054	0.622575708	2.481013618

CebHLH88	JL000551	3.73153328	2.443921351	1.03895436	0.804782727
CebHLH89	JL015227	0.275839554	1.278377386	1.02070804	1.08789693
CebHLH91	JL025286	0	0.046876824	0	0.127007586
CebHLH92	JL003085	0.037245304	0	0	0
CebHLH94	JL003888	0.866570768	0.879834975	0.712641193	0.430408706

Table S4. Primers of qRT-PCR

Gene name	Gene ID	Forward primer (5'-3')	Reverse primer (5'-3')
GAPDH	JL008987	GCAATCACCGCCACTCAGAAGA	CACGGAACGCCATACCAGTCAAT
CebHLH13	JL010983	GTTGGTTCCTTTGGCGTTGA	CATTCTCTGTGGCGTCTTCG
CebHLH75	JL019154	ACATCAAGACTCGGAAGACCAC	TCAGGAGATAATGAAGCAGAAGG

Table S5. Primers of subcellular localization

Gene name	Gene ID	Forward primer (5'-3')	Reverse primer (5'-3')
CebHLH13	JL010983	acgggggactcttgaccatggATGGCGGTAGATATGCAGAGC	aagttcttctcctttactagtTTTCACTAGTGAATCACGGCACCC
CebHLH75	JL019154	acgggggactcttgaccatggATGGCATCTGAGATGCAGACC	aagttcttctcctttactagtAAACAGATTGATCTGTCCTTTG
